# A novel *smoothed (SMO)* point mutation in congenital tibial hemimelia: a case report

**DOI:** 10.1186/s12887-023-04167-y

**Published:** 2023-08-25

**Authors:** Xiaodong Yang, Siyu Pu, Bo Xiang, Xueyang Tang, Jing Chen

**Affiliations:** 1 Department of Pediatric Surgery, West China Hospital, Sichuan University, #37 Guo-Xue-Xiang, Chengdu, 610041 China; 2 Laboratory of Pediatric Surgery, West China Hospital, Sichuan University, Chengdu, 610041 China

**Keywords:** Tibial hemimelia, Congenital anomaly, *SMO*, Polydactyly

## Abstract

**Background:**

Congenital tibial hemimelia (CTH [MIM: 275220]) is a rare congenital limb deficiency that manifests as a shortened, curved, dysplastic or absent tibia with polydactyly. In previous studies, mutations of a distant sonic hedgehog (*SHH*) cis-regulator (*ZRS*) and a Shh repressor (GLI3) were identified.

**Case presentation:**

Here, we admitted a 20-month-old boy who manifested with right tibial deformity, varus foot, ankle dislocation, and ipsilateral preaxial polydactyly. After genetic sequencing and data analysis, the results revealed a 443 A > G mutation in the father and a 536 C > T mutation in the mother in exon 2 of the *Smoothed (SMO)* gene at 7q32.1, with the coexistence of both mutant alleles in the proband/patient.

**Conclusions:**

Our report suggests that even though not previously reported, *SMO* mutations may be associated with limb anomalies such as tibial hemimelia via Hh signaling in humans and has implications for genetic counseling.

**Supplementary Information:**

The online version contains supplementary material available at 10.1186/s12887-023-04167-y.

## Background

Tibial hemimelia is a severe limb deformity with an extremely low incidence rate, with approximately one in one million (1/1000,000) live births [1, 2]. It mainly presents with a shortened, curved, dysplastic or absent tibia, a normal but usually proximal and/or distal dislocation of the fibula, varus ankle, and often polydactyly. According to previous reports, tibial hemimelia is associated with the zone of polarizing activity regulatory sequence (*ZRS*) and *GLI3* mutations [3, 4].

*Smoothed (SMO)* gene, located at 7q32.1, is evolutionarily conserved among mammals and encodes a G protein-coupled receptor that interacts with the patched protein, a receptor for Hh proteins [5]. Previous studies have indicated that the *SMO* gene plays important roles in tumorigenesis via sonic hedgehog (Shh) signaling [6]. Here, we report a case manifested with right tibial hemimelia harboring the 443 A > G and 536 C > T mutations in *SMO*. To our knowledge, this is the first case report of tibial hemimelia associated with *SMO* mutation. We present the following case in accordance with the CARE reporting checklist.

## Case presentation

**Fig. 1:**
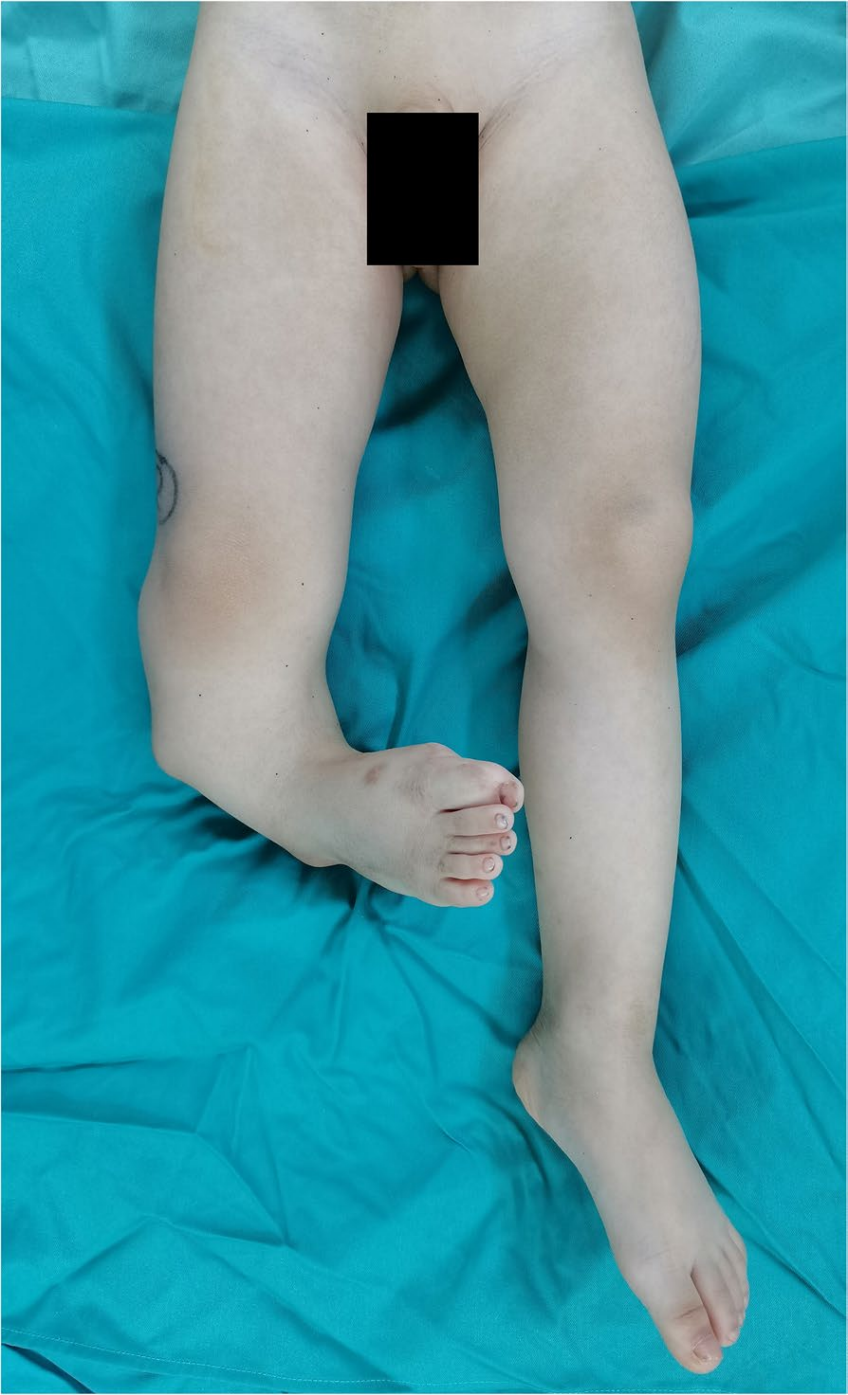
The patient’s right tibia was significantly anterolateral angulated, and approximately 8.0 cm shorter than the left, and the right foot was internal rotated. The photo was taken after the polydactylotomy

We admitted a 20-month-old boy with an abnormal right lower leg morphology since birth, accompanied by complex ipsilateral preaxial polydactyly. Consent for publication of the data was obtained from the family before the start of the study. There was no pain in the child’s right leg, normal growth in height and weight, and no abnormalities found in other parts of the body, such as the skin, hair, and face (Fig. 1). There were no abnormalities in the mother’s pregnancy and delivery and no other similar conditions in other family members. As he grew, the abnormalities of his right lower limb gradually worsened. At the time of his clinic visit, the patient had not yet learned to walk but was able to crawl normally. Physical examination revealed that the right tibia was anterolateral angulated and approximately 8.0 cm shorter than the left tibia. The right foot was internally rotated, with limited abduction and no dorsal extension at all. There was complex polydactyly on the lateral aspect of toe 1, and those toes could not move.

**Fig. 2:**
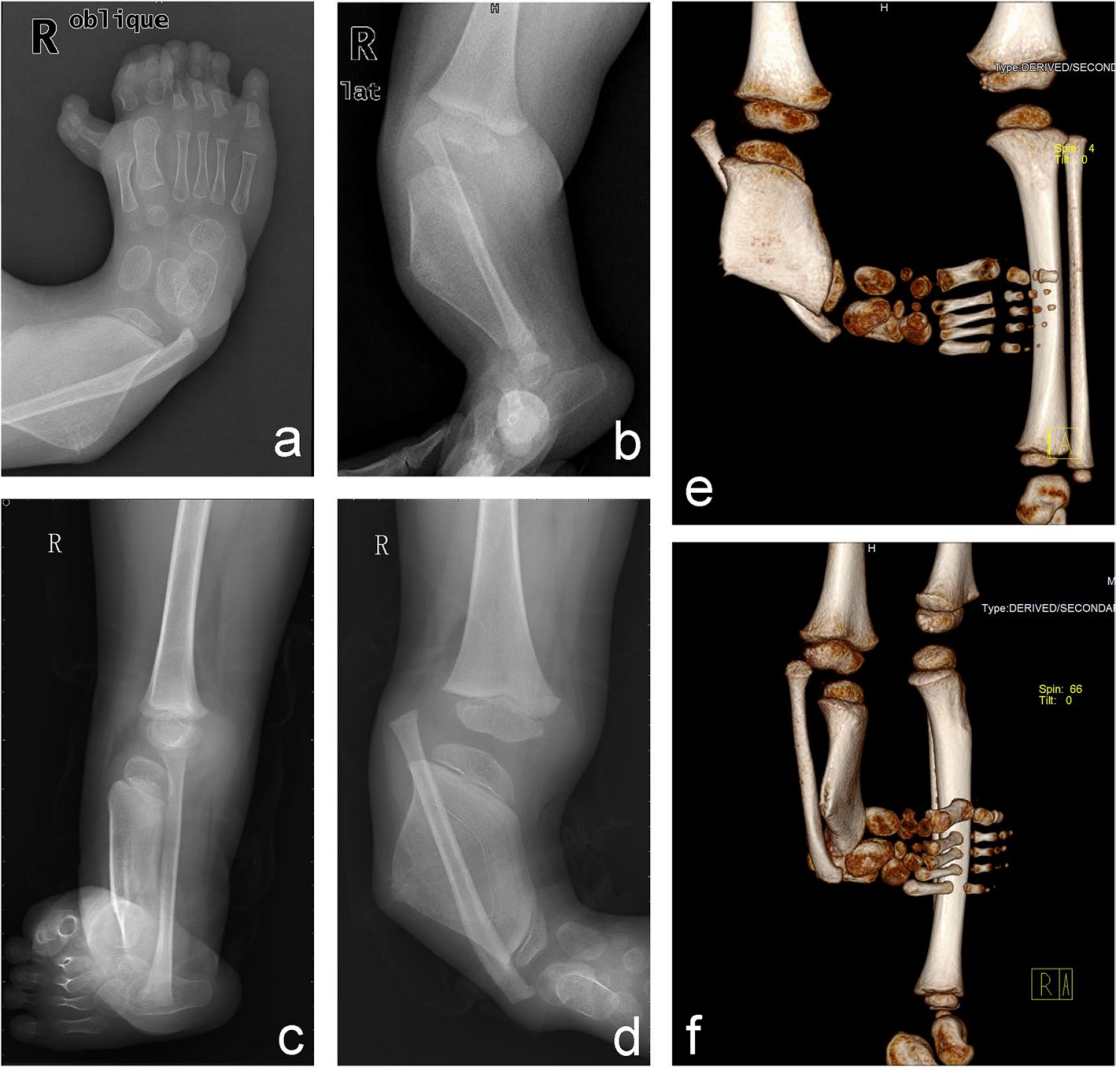
Radiological features of the proband. **a** X-ray showed polydactyly of the right toes, and a redundant metatarsal forming an articulation with the superfluous toes. **b**-**f** X-ray and computed tomography showed shortening, widening and thinning of the tibial stem, slender fibula, as well as dislocation of the and ankle

Radiological examination of the right leg revealed shortening, widening and thinning of the tibial stem, slender fibula, dislocation of the ankle, complex polydactyly of the right toes, and a redundant metatarsal forming an articulation with the superfluous toes (Fig. 2). A complete blood count, urine, blood biochemistry, chest radiograph, and ECG were within normal limits.

The child underwent polydactylotomy and right tibiofibular osteotomy reconstruction in our hospital. During the operation, we found that the morphology of the right tibia was changed from “tubular bone” to “flat bone”, and the medial margin of the tibia had a cartilage-like structure with localized thickened periosteum. The medullary cavity was not visible after cutting up the midshaft of the right tibia, but the bone hardness was basically normal. The prognosis of the child after surgery was favorable, and he is now able to stand on his own after 8 months of follow-up.

After searching the published reports, we considered this child’s case to be tibial hemimelia, which might be associated with genomic mutations. After obtaining informed consent (protocol conformed to the ethical guidelines of the 1975 Declaration of Helsinki and approved by the Institutional Review Board and Ethical Committee at the West China Hospital of Sichuan University in China), we collected peripheral blood samples from the proband, his father and mother, extracted DNA, and performed next-generation whole exome sequencing (NGS) using the Illumina platform.

## Data analysis and verification

NGS data were analyzed by Novogene (Tianjin, China). Briefly, sequencing results were aligned to the human genome (GRCh37/hg19) for SNP/INDEL calling: single nucleotide variants (SNVs) and indels were called with samtools to generate gVCF [7]. The copy number variants (CNVs) and structural variants (SV) were detected with Control-FREEC (v9.1) and LUMPY (v0.2.13) software, respectively. Then, annotation was performed using ANNOVAR (2017June8) [8]. Filtering of rare variants was performed as follows: (1) variants with an MAF less than 0.01 in 1000 genomic data (1000g_all) [9], esp6500siv2_all [10], gnomAD data (gnomAD_ALL and gnomAD_EAS) [11] and in the house Novo-Zhonghua exome database from Novogene; (2) only SNVs occurring in exons or splice sites (splicing junction 10 bp) were further analyzed since we are interested in amino acid changes. (3) Then, synonymous SNVs that were not relevant to the amino acid alternation predicted by dbscSNV were discarded. Small fragment nonframeshift (< 10 bp) indels in the repeat region defined by RepeatMasker were discarded. (4) Variations were screened according to scores of SIFT [12], Polyphen [13], MutationTaster [14] and CADD [15] software. The potentially deleterious variations are reserved if the score of more than half of these four software programs supports the harmfulness of variations [16]. Sites (> 2 bp) that did not affect alternative splicing were removed. To better predict the harmfulness of variation, the classification system of the American College of Medical Genetics and Genomics (ACMG) was used. The variations are classified into pathogenic, likely pathogenic, uncertain significance, likely benign and benign [17]. Then, variant-phenotype associations were analyzed with Phenolyzer and DisGetNet (V5.0). In our case, the SMO gene with two compound heterozygous variants (NM_005631: c.C536T:p.T179M and c.A443G:p.Q148R) was one of 25 genes in the candidate gene list found with Phenolyzer. Moreover, the SMO gene was the only gene found in DisGetNet associated with the phenotype of tibial hemimelia/polysyndactyly.

**Fig. 3:**
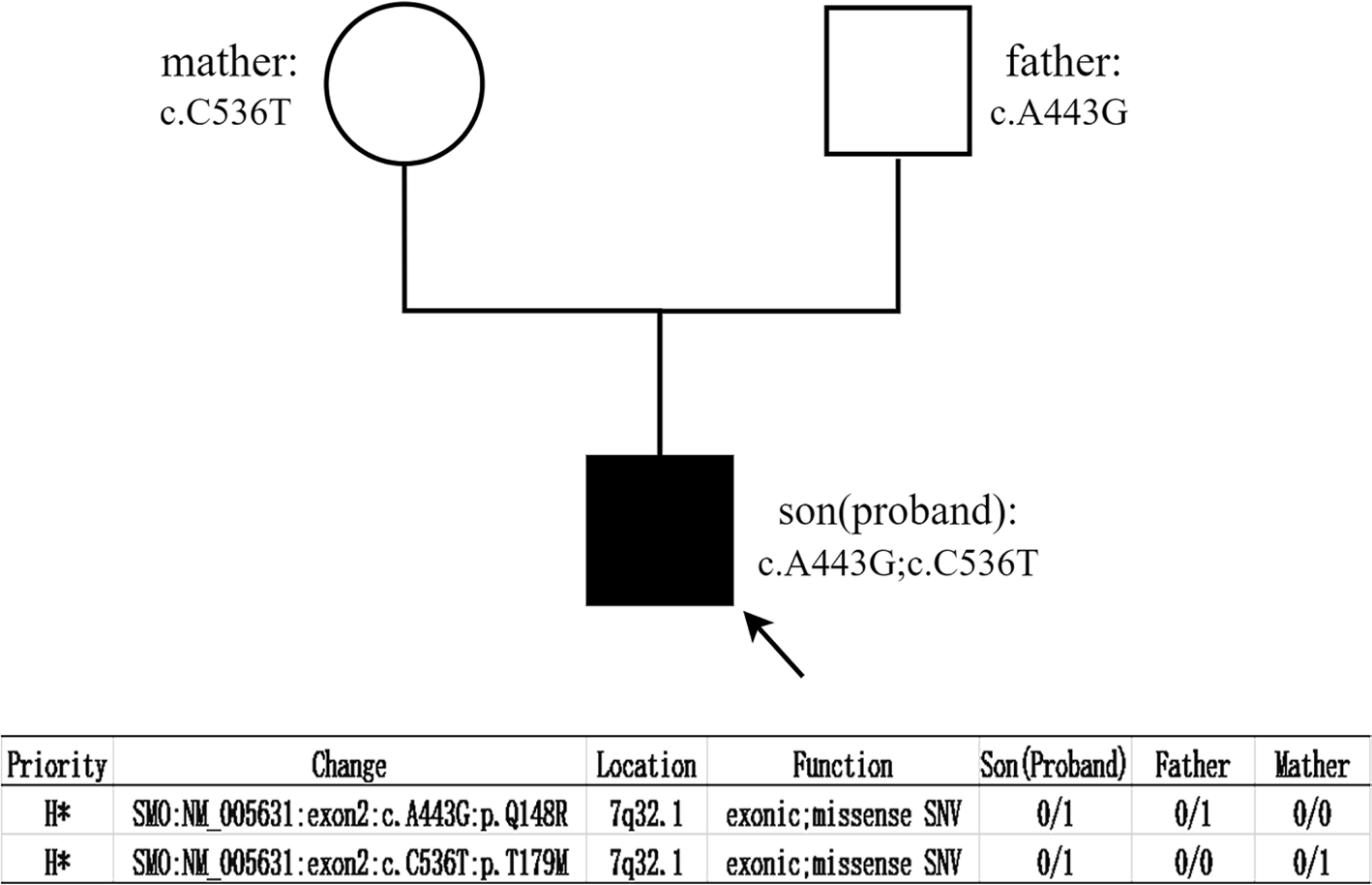
Pedigree of the family with of tibial hemimelia and the genetic sequence results

**Fig. 4:**
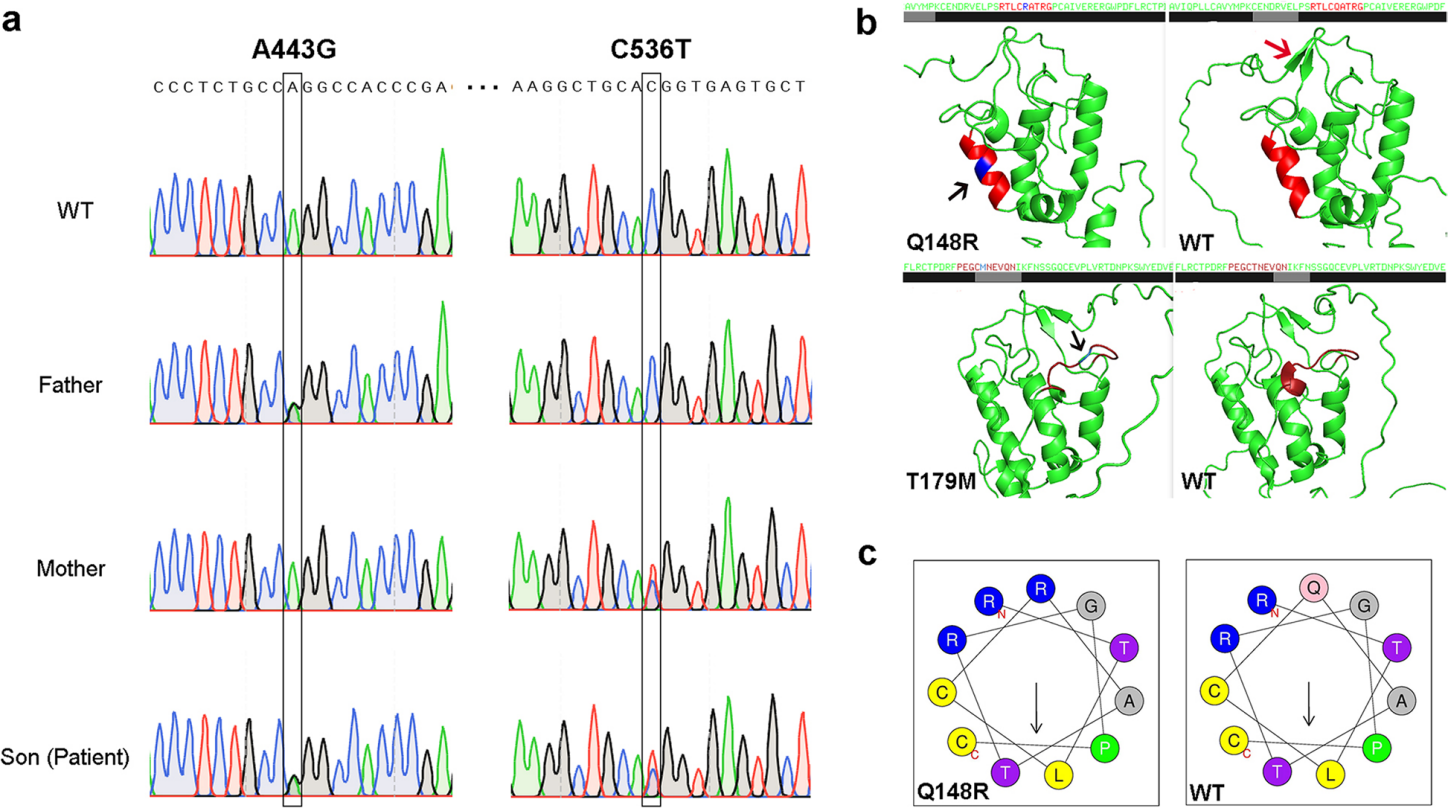
Mutation validation and protein structure modeling. **a** PCR sequencing result showing a heterozygous A443G mutation of *SMO* in the father and C536T in the mother, while compound heterozygous in the proband, but none in the wildtype (WT). **b** Both mutations are located in the CRD/Fz domain, which may potentially disrupt the protein/ligand or sterols binding affinity of SMO receptor. The black arrow indicates the mutation site and the red arrow indicates the presence of β-folding in WT but not in Q148R. the protein structures were simulated by pymol softwere. **c** Q148R substitution will change the local charge and polarity of the Helix structure, which was ploted with HeliQuest

Based on the above analysis, we initially determined that the father and mother in this family each had a point mutation in exon 2 of the *SMO* gene at 7q32.1, and both missense mutations (father: *SMO*: NM_005631:c.A443G:p.Q148R or NC_000007.13:g. 128843336A > G; mother: SMO: NM_005631:c.C536T:p.T179M or NC_000007.13:g.128843429 C > T) and present in the child as compound heterozygous mutation, thus resulting in the disease (Fig. 3). Then, we designed primers (forward primer 5’-CTAGCAGGGCATCTGGAAGT-3’ and reverse primer 5’-TATACCCGGTCCTGCCCAAC − 3’) to amplify a 771 bp fragment covering these two mutation sites to validate the NGS result. Genomic DNA of peripheral blood from the father, mother, son (patient) and a wild type were applied to polymerase chain reaction, and amplified products were used for Sanger sequencing. The sequencing results showed a heterozygous 443 A > G mutation in the father, 536 C > T in the mother, and both in the proband but not in the normal control. This result verified the NGS findings (Fig. 4).

## Discussion and conclusion

The prevalence of limb anomalies in neonates is approximately 0.38% in China, with severe cases significantly affecting the survival and long-term quality of life of patients, most of whom require multiple operations [18, 19]. The malformations of tibial hemimelia may be unilateral or bilateral, and the tibia may be shortened, curved, dysplastic or even absent, along with various other limb deformities [20, 21]. There are different treatments for tibial hemimelia depending on the severity, and amputation is the most tried and proven method and is currently considered the gold standard of treatment for tibial hemimelia [20]. In our case, however, although the child’s shortened and “flattened” tibia had resulted in ankle dislocation and complete loss of standing ability, the parents could not accept amputation, and he ultimately underwent three operations for reconstructing the right lower limb structure and restoring function.

Tibial hemimelia is the result of abnormal limb bud development [22]. During embryonic development, bone formation is regulated by various signaling molecules, among which Hh is strongly involved. The Hh protein in mammals has three homologs, Shh, Indian hedgehog (Ihh) and Desert hedgehog (Dhh) [23]. At early stages of embryonic development, Shh regulates the formation of the zone of polarizing activity (ZPA) as a morphogen to establish the anterior-posterior (A-P) patterning of the embryonic limb bud [24, 25]. Ihh participates in the process of endochondral bone formation at late stages of embryonic development together with thyroid hormone-related peptide (PTHrP), which regulates growth plate and long bone development [26]. Previously reported tibial hemimelia associated with mutations in *GLI3* and *ZRS* further validate the importance of the Shh signaling pathway in limb bud development [3, 4]. In addition to *GLI3* and *ZRS*, Shh signaling is also regulated by the seven transmembrane protein SMO [27, 28].

SMO protein plays a key role in transducing Hh signaling. When the Hh ligand binds to the patched protein, the patched-mediated inhibition of SMO is released, allowing the signal to be transduced [29]. Although no limb abnormalities have been reported to be associated with *SMO* mutations, as mentioned above, it is speculated that *SMO* may also be involved in regulating limb development through the Hh signaling pathway. This case had two point mutations in exon 2 of the *SMO* gene at 7q32.1, and both were missense, which will cause a single amino acid substitution instead of a change in protein length. The SMO protein is an integral membrane receptor with an extracellular cysteine rich domain (CRD), also called the Fz domain, a transmembrane domain and an intracellular domain. Both of these mutations are located in the CRD/Fz domain (65-181Aa), which may potentially disrupt the protein/ligand or sterol binding affinity of the SMO receptor. For the maternally derived mutation (c.536 C > T;p.T179M;rs115491500) according to the ACMG criteria is classified as “likely pathogenic”; nevertheless, the paternal mutation (c.443 A > G;p.Q148R) is classified as of “uncertain significance”. However, Q148 is located in a Helix motif formed by 8 amino acids (144-RTLCQATR-151; PDB: 5V56), and the Q > R substitution will change the local charge and polarity, leading to confirmation change and structural disturbance (Fig. 4b and c). Moreover, this Q148R substitution may somehow affect disulfide bond formation between amino acids 147 and 156 C (147-CQATRGPCAIVERERGWPDFLRC-156; UniRule PROSITE-ProRule: PRU00090). Thus, we speculate that the structure or function of the SMO protein may have been disrupted in our case, affecting Hh signal transduction and leading to the malformation condition of the proband.

In conclusion, we report a case of tibial hemimelia that may be associated with point mutations in the *SMO* gene. The finding could help provide targeted gene sequencing technology to improve the pregnancy screening of this family and fetuses with similar presentations. The data also suggest that SMO mutations may also be involved in the development of limb abnormalities in humans, and further studies are needed to confirm this, potentially improving the process of genetic studies of limb development.

### Supplementary Information

**Additional file 1.**

## Data Availability

All data generated or analysed during this study are included in this published article.

## Abbreviations

SMO: Smoothed
Shh: Sonic hedgehog
ZRS: Zone of polarizing activity regulatory sequence
Ihh: Indian hedgehog
Dhh: Desert hedgehog
ZPA: Zone of polarizing activity
A-P: Anterior-posterior
PTHrP: Thyroid hormone-related peptide
