# Supplementary material for: A novel smoothed (SMO) point mutation in congenital tibial hemimelia: a case report
Source: BMC Pediatr. 2023 Aug 25;23:424. doi: 10.1186/s12887-023-04167-y (PMC10463978; doi:10.1186/s12887-023-04167-y)
Supplement: Supplementary file 1 — Additional file 1. [file 12887_2023_4167_MOESM1_ESM.pdf]

CARE case report guidelines

CARE Checklist of information to include when writing a case report

(cc) BY-NC-ND

☑

Topic	Item	Checklist item description	Reported on Line
Title	1	The diagnosis or intervention of primary focus followed by the words "case report"	page 1 line 1
Key Words	2	2 to 5 key words that identify diagnoses or interventions in this case report, including "case report"	page 2 line 34
Abstract (no references)	3a	Introduction: What is unique about this case and what does it add to the scientific literature?	page 2 line 7-12; 23-28
	3b	Main symptoms and/or important clinical findings	page 2 line 12-15
	3c	The main diagnoses, therapeutic interventions, and outcomes	page 2 line 17-23
	3d	Conclusion—What is the main "take-away" lesson(s) from this case?	page 2 line 23-28
Introduction	4	One or two paragraphs summarizing why this case is unique (**may include references**)	page 3 line 7-31
Patient Information	5a	De-identified patient specific information.	page 3 line 37-52
	5b	Primary concerns and symptoms of the patient.	page 3 line 37- 39
	5c	Medical, family, and psycho-social history including relevant genetic information	page 3 line 46- 52
	5d	Relevant past interventions with outcomes	page 3 line 48-52
Clinical Findings	6	Describe significant physical examination (PE) and important clinical findings.	page 3 line 52-58
Timeline	7	Historical and current information from this episode of care organized as a timeline	page 4 line 7-17
Diagnostic Assessment	8a	Diagnostic testing (such as PE, laboratory testing, imaging, surveys).	page 3 line 60; page 4 line1-5
	8b	Diagnostic challenges (such as access to testing, financial, or cultural)	page 4 line 19-61; page 5 line1-19
	8c	Diagnosis (including other diagnoses considered)	page 5 line 21-44
	8d	Prognosis (such as staging in oncology) where applicable	page 4 line 15-17
Therapeutic Intervention	9a	Types of therapeutic intervention (such as pharmacologic, surgical, preventive, self-care)	page 4 line 7-31
	9b	Administration of therapeutic intervention (such as dosage, strength, duration)	page 4 line 7-15
	9c	Changes in therapeutic intervention (with rationale)	page 4 line 19-31
Follow-up and Outcomes	10a	Clinician and patient-assessed outcomes (if available)	page 4 line 16-17
	10b	Important follow-up diagnostic and other test results	page 5 line 21-44
	10c	Intervention adherence and tolerability (How was this assessed?)	page 4 line 21-27
	10d	Adverse and unanticipated events	not available
Discussion	11a	A scientific discussion of the strengths AND limitations associated with this case report	page 6 line 27-61; page 7 line 1-9
	11b	Discussion of the relevant medical literature **with references**.	page 5 line 50-61; page 6 line 1-36
	11c	The scientific rationale for any conclusions (including assessment of possible causes)	page 6 line 9-36
	11d	The primary "take-away" lessons of this case report (without references) in a one paragraph conclusion	page 7 line 11-21
Patient Perspective	12	The patient should share their perspective in one to two paragraphs on the treatment(s) they received	page 4 line 7-17
Informed Consent	13	Did the patient give informed consent? Please provide if requested	Yes ☑ No ☐
